# The Polygenic Map of Keloid Fibroblasts Reveals Fibrosis-Associated Gene Alterations in Inflammation and Immune Responses

**DOI:** 10.3389/fimmu.2021.810290

**Published:** 2022-01-10

**Authors:** Yang Li, Min Li, Caijie Qu, Yongxi Li, Zhanli Tang, Zhike Zhou, Zengzhao Yu, Xu Wang, Linlin Xin, Tongxin Shi

**Affiliations:** ^1^ Department of Dermatology, The Affiliated Qingdao Municipal Hospital of Qingdao University, Qingdao, China; ^2^ Department of Dermatology, Qilu Hospital, Shandong University, Jinan, China; ^3^ Department of Dermatology, Shandong Provincial Qianfoshan Hospital, The First Hospital Affiliated with Shandong First Medical University, Jinan, China

**Keywords:** fibroblasts, keloid, molecular alterations, genome-wide expression, immune response, inflammation

## Abstract

Due to many inconsistencies in differentially expressed genes (DEGs) related to genomic expression changes during keloid formation and a lack of satisfactory prevention and treatment methods for this disease, the critical biomarkers related to inflammation and the immune response affecting keloid formation should be systematically clarified. Normal skin/keloid scar tissue-derived fibroblast genome expression data sets were obtained from the Gene Expression Omnibus (GEO) and ArrayExpress databases. Hub genes have a high degree of connectivity and gene function aggregation in the integration network. The hub DEGs were screened by gene-related protein–protein interactions (PPIs), and their biological processes and signaling pathways were annotated to identify critical biomarkers. Finally, eighty-one hub DEGs were selected for further analysis, and some noteworthy signaling pathways and genes were found to be closely related to keloid fibrosis. For example, IL17RA is involved in IL-17 signal transduction, TIMP2 and MMP14 activate extracellular matrix metalloproteinases, and TNC, ITGB2, and ITGA4 interact with cell surface integrins. Furthermore, changes in local immune cell activity in keloid tissue were detected by DEG expression, immune cell infiltration, and mass CyTOF analyses. The results showed that CD4+ T cells, CD8+ T cells and NK cells were abnormal in keloid tissue compared with normal skin tissue. These findings not only support the key roles of fibrosis-related pathways, immune cells and critical genes in the pathogenesis of keloids but also expand our understanding of targets that may be useful for the treatment of fibrotic diseases.

## Introduction

A keloma, also known as a keloid, is a benign dermal collagen tumour caused by chronic skin tissue lesions and inflammation that mainly forms due to collagen and extracellular matrix (ECM) deposition (1, 2). The development of keloids has a tendency to recur but does not subside. It usually occurs in families which indicates the genetic background of the disease (3). Although keloids are not life-threatening, the incidence and recurrence of this disease may be high, and this type of wound healing disorder seriously affects quality of life due to pain, itching, and restricted mobility (4). In addition, the main reason for keloid formation has not been clearly determined, and no satisfactory treatment method is available (5).

Some studies have used microarray technology to compare fibroblast cultures of normal skin/keloid scar tissues. These genome expression studies have found many genes and pathways related to keloid fibrosis diseases, such as hypoxia inducible factor-1α (6), insulin-like growth factor binding protein-3 and the Wnt signaling pathway (7). Microarray studies have also identified common patterns of gene expression changes in autoimmune diseases (8). However, these studies all included small samples, and considerable heterogeneity was identified between the results for gene expression differences. To accurately screen the key genes and pathways in keloid formation and develop procedures to alter genes related to fibroblasts in lesions, genome aggregation analysis was applied to improve statistical analysis capabilities and the credibility of the results. In this study, five gene expression profiles including 29 keloid and 24 normal specimen tissues, were acquired from the public Gene Expression Omnibus (GEO) and ArrayExpress databases, and the genome-wide expression profiles of normal skin/keloid scar fibroblasts were comprehensively analyzed.

## Materials and Methods

### Data Acquisition and Standardization

The public GEO and ArrayExpress genome databases were searched using the keyword “Keloid”, and genome-wide expression data for cultured fibroblast samples isolated from normal skin/keloid scar tissue were screened. Background denoising and standardization of gene chip data are important prerequisites for exploring changes in gene expression levels. In this study, the “Aquantile” method of package limma (version 3.40) in R-4.0.4 software was used to perform background and standardization processing on the chip data.

### Gene Expression Differential Analysis

The Wald test and Benjamini-Hochberg method were used to identify differentially expressed genes (DEGs) in keloids, and P values (P <0.05) were calculated to identify differentially expressed genes with significant changes. To reduce differences in results between different sequencing platforms and studies, first, DEGs between normal and keloid tissue fibroblasts from each study were obtained separately. Then, the interset operation was used to classify the differentially expressed genes obtained in each group, and the change in gene expression was used as the weight analysis factor for each differentially expressed gene to further screen potentially important genes.

### Cellular Immune Infiltration Analysis

According to Bindea’s (9) research results on 28 immune cell types, with typically altered genes as the background, enrichment score analysis of the gene dataset in this study was carried out by GSVA (R package GSVA, version 1.32, Gene Set Variation Analysis: Estimates GSVA enrichment scores) (10). The immune scores for stromal/immune cells present in tissues were predicted through the R package estimate, version 1.0. Pearson correlations for the immune scores from estimates and enrichment scores from GSVA were applied to explore discrepancies in immune cell function between normal skin/keloid scar tissue.

### DEG-Related Protein Interaction Analysis

Interactions between cell molecules are a key factor affecting the occurrence and recurrence of keloids. Therefore, to identify important DEGs in keloid fibroblasts, interactions between important DEG-related proteins (including transcription factors) were analyzed to further understand molecular biological changes in keloid fibroblasts. In this study, based on the STRING v11.0 (https://string-db.org/cgi/input.pl) database (11), the protein interactions of the DEGs of the GEO samples were analyzed according to a parameter score threshold > 400 and a degree > 2 in the *STRINGdb* package (version 2.3.0).

### Gene Ontology and Pathway Analysis

The gene ontology and signaling pathways involved in key DEGs are of great significance to the process of keloid formation. This study used gene ontology (GO) and Kyoto Encyclopedia of Genes and Genomes (KEGG) in clusterProfiler (12) to analyse relationships between key genes, the biological processes of keloid fibroblasts, and immune pathway activities, including fibroblast activation, fibronectin binding, and immune cell activation and regulation.

### Flow Cytometric Detection of Local Tissue Immune Cell Types

Keloid and normal skin tissues were obtained with patient consent. The tissues were cut into small particles (Φ1~2 mm) with scissors, rinsed with calcium- and magnesium-free PBS, placed in a petri dish with subsequent addition of 30 times the tissue enzyme solution (0.1~0.3 μg/ml collagenase), placed in a 37°C water bath or incubator shaker, and digested for 20–60 min. Then, serum-containing medium was added to terminate digestion, followed by centrifugation at 300 g for 5 min and then washing with buffer such as PBS 1~2 times. Dyed antibodies were provided by Abcam and Invitrogen suppliers (**SupplementaryTable S5**). With the noise reduction mode off, CyTOF data were obtained by a CyTOF2 instrument (Zhejiang Proting Health Technology Co., Ltd.) and analyzed using the dual count mode (calibrated in flight, combined with the pulse count and strength information). Finally, the FCS file was analyzed by FlowJo vX (Tree Star, Inc., Ashland OR) and R packages *flowCore* (version 2.2.0) and *CATALYST* (version 1.14.0).

## Results

### Differentially Expressed Genes in the Data Sets

A total of 5 sets of keloid gene expression microarray data were used for further analysis. The GEO datasets were GSE7980 (7), GSE44270 (13), and GSE145725 (6), and the ArrayExpress datasets were E-MTAB-2509 (14) and E-MTAB-4945 (**Table 1**).

**Table 1:** Data sets select and design information.

Author (Year)	Dataset(Probes)	Chip/Sample Nums	Overall design	Article DOI
Hahn et al. (13) Rebekah.Karns@cchmc.org	GSE44270 (28869)	KFs: GSM1081582; GSM1081583; GSM1081584; GSM1081585; GSM1081586; GSM1081587; GSM1081588; GSM1081589; GSM1081590; NFs: GSM1081608; GSM1081609; GSM1081610	Skin and scar tissues were obtained for isolation of primary fibroblasts. Nine keloid scars and three normal skin samples were obtained and cultured. RNA was isolated using RNeasy, and quality verified using an Agilent 2100 Bioanalyzer. Labeling and hybridization to **Affymetrix Human Gene 1.0 ST** microarray chips was performed by the Vanderbilt Genome Sciences Resource at Vanderbilt University Medical Center.	10.1111/wrr.12060
Smith et al. (7) shirley.b.russell@vanderbilt.edu	GSE7890 (54675)	KFs: GSM194109; GSM194110; GSM194111; GSM194112; GSM194113; NFs: GSM194118; GSM194119; GSM194120; GSM194121; GSM194122	Cell cultures of fibroblasts were initiated from human biopsy material from 5 normal dermal scars and 5 keloids of adult males and females. Experimental cultures were derived from the first passage of cells thawed from liquid nitrogen. RNA from each cell strain was isolated from three independent cell cultures and pooled, then run on an **Affymetrix Human Genome U133 Plus 2.0** GeneChip.	10.1038/sj.jid.5701149
Kang et al. (6) HTSAO@mgh.harvard.edu	GSE145725 (49495)	KFs: GSM4331585; GSM4331586; GSM4331587; GSM4331588; GSM4331589; GSM4331590; GSM4331591; GSM4331592; GSM4331593; NFs: GSM4331594; GSM4331595; GSM4331596; GSM4331597; GSM4331598; GSM4331599; GSM4331600; GSM4331601; GSM4331602; GSM4331603	Briefly, 10 cell lines isolated from patients with 19 samples (10 NFs and 9 KFs) were subjected to RNA extraction using the Qiagen RNeasy Mini Kit (Qiagen). RNA samples with RNA integrity number (RIN) above 9.8 were hybridized to **GeneChip PrimeView Human Gene Expression Arrays (Affymetrix),** and analyzed using GeneChip Expression Console.	10.1016/j.jid.2020.01.036
Wong et al. (14) potteram@ohsu.edu	E-MTAB-2509 (24535)	NFs: Chip 1; Chip 2; Chip 3; KFs: Chip 19; Chip 20; Chip 21	Harvested fibroblasts from 3 normal skin and 3 Keloids. Hybridization reagents were mixed with 750 ng of labeled RNA, and the mixture was hybridized overnight at 58 °C to **HumanRef-8 v3.0 Expression BeadChips™ array (Illumina^®^)**.	10.1016/j.jss.2013.04.006
Leo Zeef (53) Leo.Zeef@manchester.ac.uk	E-MTAB-4945 (50683)	NFs: Sample 23; Sample 25; Sample 26; KFs: Sample 24; Sample 27; Sample 28	There were 3 of each normal and keloid tissue used for microarray. RNA extracted using Qiagen RNeasy Micro Kit. Extracted RNA was amplified using the Ovation^®^ Pico WTA system v2 kit (NuGen Technologies) and purified with QIAquick PCR purification kit (Qiagen). **SurePrint G3 Human GE 8x60K V2** were scanned using an Agilent Microarray Scanner.	NA

KF, keloid fibroblasts; NF, normal skin/scar fibroblasts; NA, not available.

Gene expression differences in keloid fibroblasts in each study group were displayed in **Table 2**, but the expression difference in each gene was heterogeneous among the different groups, thus necessitating examination of the discrepancies in the expression of important genes. In this study, we first used the intersecting relationship to analyse the distributions of DEGs. The results showed that these five datasets had only three common genes: BPGM, SKAP2, and NEO1 (**Supplementary Figure S1A**). We found that the expression levels of these genes in the GSE145725 group were opposite to those in the other four groups (**Supplementary Figure S1C**), which contained 5 common genes: NEO1, SKAP2, BPGM, RCC2, and TMEM120A.

**Table 2:** DEGs of GEO and AarryExpress datasets.

	Genome ID	Total (Up/Down) DEGs	Shared DEGs (Up/Down/divergent)	Top significant pathways*
GEO	GSE145725	3951 (2065/1886)	**124(18/12/94)** (SIX1, MAK16, LRP4, APBA2, CA12, HOXA9, RGS4, TBX15, RIPOR2, HOXA10, HOXC4, ANO6, CCDC80, GABBR2, HOXC9, RHPN2, SGMS2, STMN2, HOXB7, TSHZ1, CES1, OSBPL8, BPGM, TMEM204, UGCG, RAB20, HOXA5, CCPG1, HSPA2, SERPINB7, PPP3CC, TNFAIP1, SKAP2, PAFAH1B3, USP53, TNC, FKBP1B, ARRDC4, HDAC1, SOCS6, ECHDC2, PRSS12, DIRAS3, EPHB2, SLFN11, PFKP, SIX4, TRPV2, GPR37, AKR1C2, NKRF, HSPB8, BMPER, KCNS3, FHL1, SRPX, PHACTR2, CHN1, HDGFL3, GNPNAT1, SMS, CDC14B, SCD5, OXSR1, GALC, ADGRE5, ADK, ZADH2, PRKD1, SLIT2, SFRP2, PIK3R1, HOXB3, CDKN1A, F2RL2, RWDD4, CARD16, AGPAT5, HOXC5, GPN1, VEPH1, SLC7A11, METTL22, FAM3C, RESF1, SSX2IP, EIF2S2, RP9, TUFT1, SIVA1, IARS, CENPV, NMT2, NEO1, PGM1, CTSZ, SSBP3, PTGER2, SETDB1, TSC22D4, RBPJ, WDR75, CDCA7, CENPA, FAM91A1, TENM4, H2AFJ, WDR81, CHSY1, SH3BGR, GUCY1B1, EBF1, RHOBTB3, ADH1B, FOXP1, KCTD10, DAXX, CFB, PLAG1, SPAG5, TP53, NFU1, LIMS2, NOX4)	(1) Neutrophil degranulation; (2) RUNX3 Regulates Immune Response and Cell Migration; (3) Amino acid synthesis and interconversion; (4) Mucopolysaccharidoses; (5) ATF6 (ATF6-alpha) activates chaperone; (6) Glycosphingolipid metabolism; (7) Keratan sulfate degradation; (8) Post-translational protein phosphorylation; (9) Scavenging by Class A Receptors; (10) Ephrin signaling; (11) Signaling by Interleukins; (12) Regulation of Insulin-like Growth Factor (IGF) transport and uptake by Insulin-like Growth Factor Binding Proteins (IGFBPs); (13) Interleukin-17 signaling; (14) TCF dependent signaling in response to WNT; (14) Toll-like Receptor Cascades
	GSE7890	1384 (709/1303)		
	GSE44270	2856 (1489/1367)		
AarryExpress	E-MTAB-2509	1214 (626/588)	**91(37/35/19)** (TNS1, GPC1, TRIB2, TPM1, NEO1, SKAP2, BPGM, AMOT, GPSM2, PAQR5, GRIK2, NEU1, SHB, EFCAB2, RCC2, SQLE, PROS1, TMEM120A, DNAJA4, CPNE8, COQ2, F10, DDIT4L, OSR1, DBI, RASSF2, CUBN, FBLIM1, ETV6, OSBPL3, FANCE, TRAPPC2L, OXTR, CIDEC, NMNAT2, C1orf198, TNFRSF14, TRIM16L, NAV3, PSG3, TRH, SRF, CDH1, DHX58, MYL9, DPYD, DHCR7, SHROOM2, CSGALNACT1, CORIN, TMEM200A, GLS, ROGDI, CCDC102B, FAM83H, COL5A1, ALDH1B1, EGR1, MTMR9, CD109, AHNAK, APBB1IP, BMP1, DES, MTG1, DUSP22, COL3A1, CABLES1, SH3BP2, OPCML, RAP2B, CCL18, C12orf29, HCN4, NID2, CADM1, GPR3, FDFT1, TRHR, FADS1, TPP1, FAM9C, AKNA, SP5, COL1A2, ABAT, TMEM154, RPS6KA6, NCOR2, DLX5, PACRG)	
	E-MTAB-4945	1210 (636/575)		

DEGs: differential expressed genes;

*Only shows top 15 significant pathways, more information refers to **Supplementary Table S2**.

To reduce the impact of inconsistent DEGs data sets in different research groups on the results, this study lists the relationships of the following subsets: the datasets (GSE7980, GSE44270 and GSE145725) derived from the GEO database consist of 124 common DEGs, including 18 upregulated and 12 downregulated genes and 94 genes whose expression is objectionable (up- or down-regulated) (**Supplementary Figure S1B**). The datasets (E-MTAB-2509 and E-MTAB-4945) derived from the ArrayExpress database consisted of 61 common DEGs, including 37 upregulated and 35 downregulated genes and 19 genes whose expression was objectionable (**Supplementary Figure S1B**). The detailed results of common DEGs included in the subsets are shown in **Table 2**.

### Immune Cell Infiltration Focusing on T Cell Subtypes

**Figure 1:**
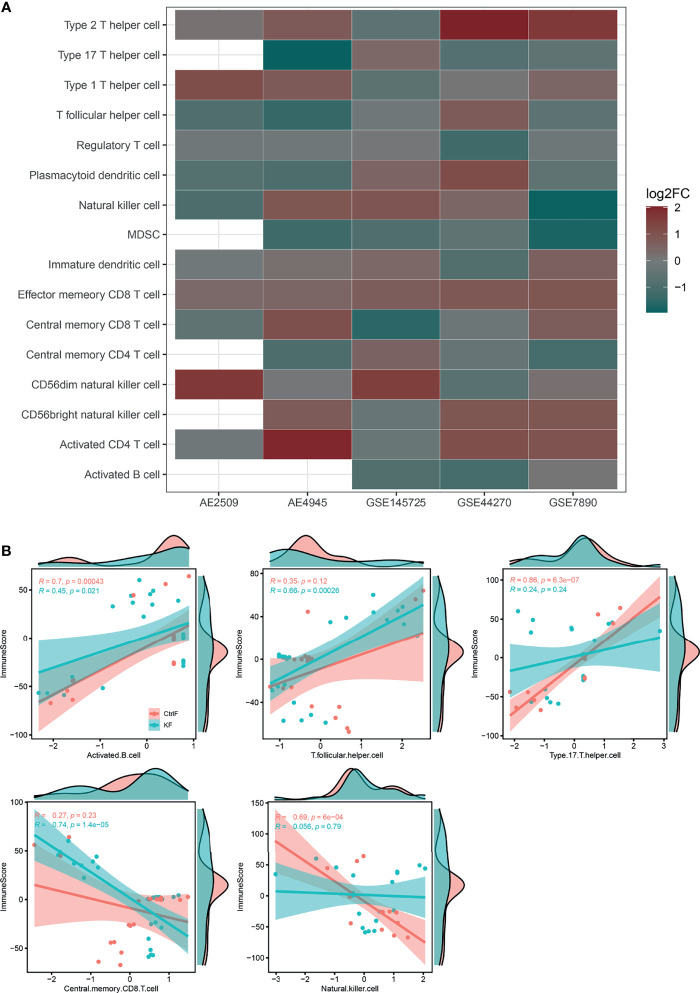
Immune cell infiltration results and correlation difference between keloid and normal fibroblast. **(A)** Bubble plot for comparison of the immune cell enriched z-scores difference between keloid and normal fibroblast. Red color indicates a higher immune cell-type z-scores in keloid fibroblast as compared with normal skin/scar fibroblast, while green color indicates a lower z-scores. The size of the circle represents the log2 fold change (log2FC) of the z-scores. **(B)** Pearson correlation of tumor estimated immune scores and immune cell GSVA z-scores of each samples between keloid and normal skin/scar fibroblast. Only shown different significant results between two group samples. Full results refer to **Supplementary Figure S1**. (AE, ArrayExpress; GEO, Gene Expression Omnibus; KF, keloid fibroblast; CtrlF, normal skin/scar fibroblast).

The immune response is the result of immune cells in the body reacting to abnormal physiological or pathological stimuli. The results of immune cell infiltration analysis showed that changes in DEGs were mainly concentrated in T cell subtypes (e.g., Th1, Th2, Th17, T-reg, CD8Tcm, CD8Tem, CD4Tcm, T follicular, and activated T cells), followed by NK cells (CD56dim NK, CD56bright NK and NK cells) (**Figure 1A**). A P value < 0.01 for immune score Pearson correlations and infiltration results for the keloid or normal tissues group was considered indicative of a significant positive(+) or negative(-) correlation. Keloid group included T follicular helper cells (+) and central memory CD8 T cells (-), while normal group included activated B cells (+), natural killer cells (-) and type 17 T helper cells (+) (**Figure 1B**). For the complete list of correlation differences between keloid and normal groups, refer to **Supplementary Figure S2**.

### Hub DEGs Filtered Through PPIs Are Related to Inflammation and the Immune Response

**Figure 2:**
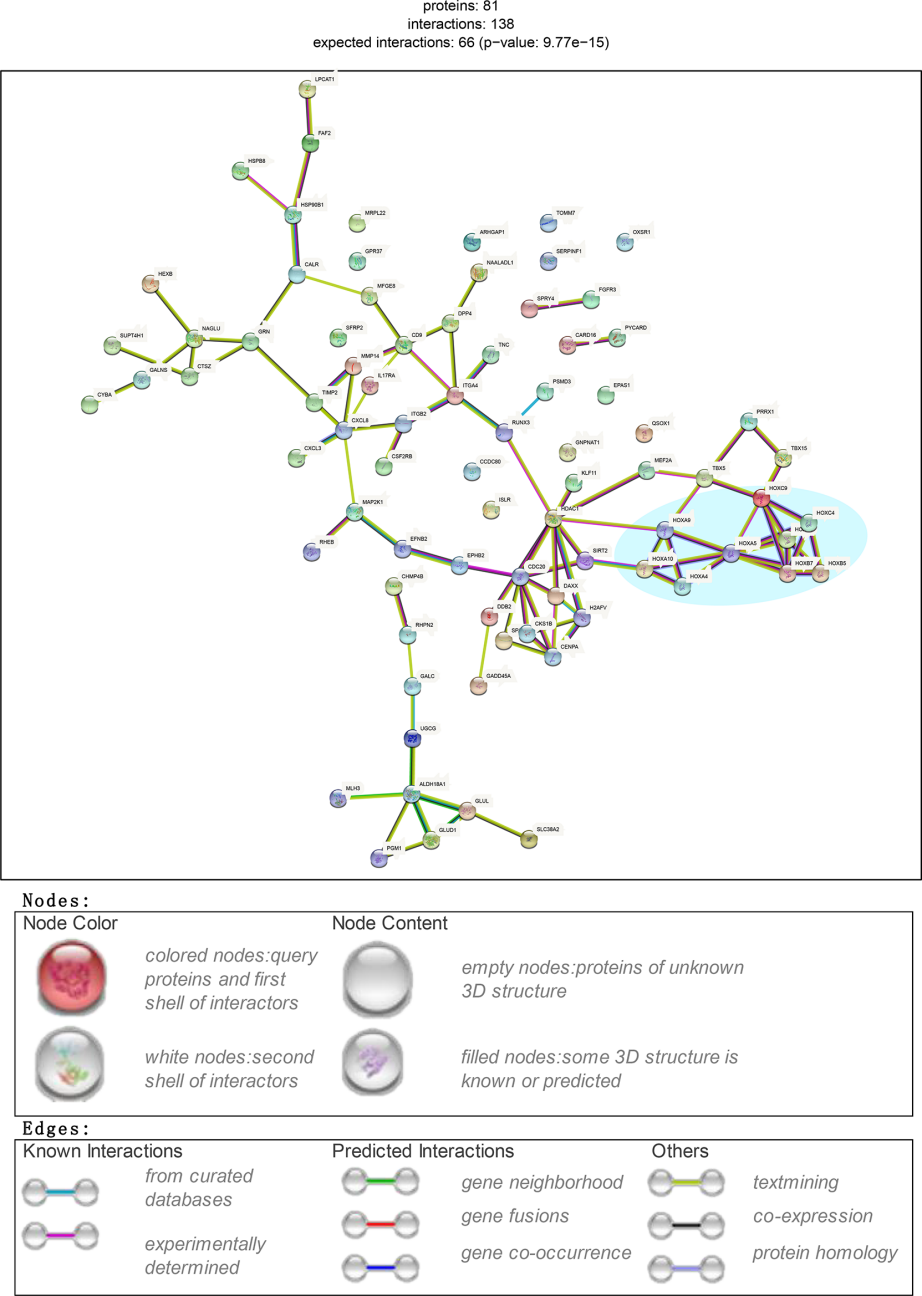
Screening critical DEGs through PPI plot. Subset DEGs with P value < 0.01. PPI applied with protein score_threshold > 400 and degree > 2.

Based on protein threshold scores (> 400) and degrees (> 2) of the PPI network, we obtained 81 core genes in the GEO datasets and rebuilt the PPI network (**Figure 2**). As found in datasets GSE44270 (13), GSE7890 (7), and E-MTAB-2509 (14), a cluster of proteins with homeodomains encoded by homeotic (HOX) genes constituted a subnetwork and played important roles in several cell types’ (such as epithelial, endothelial and myeloid cells) activation, maturation and/or differentiation according to their Gene Ontology (GO) enrichment profiles (**Supplementary Table S1**).

The biological states or processes of hub DEGs were summarized and displayed based on hallmark gene sets derived from the MSigDB database considering its specific and coherent expression data (15). A computational methodology aimed at reducing noise and redundancy was applied to retain identified overlaps and coordinate expression between gene sets in other MSigDB collections (15). Finally, the results provided a clearer understanding of the gene set enrichment profiles of these 81 hub DEGs (**Supplementary Figure S3**). As shown in the heatmap with count values corresponding to biological states or processes, inflammation and the immune response were highly aggregated.

### Multiple DEGs in Neutrophil- and T Cell-Mediated Immunity Are Reduced in Keloid Fibroblasts

**Figure 3:**
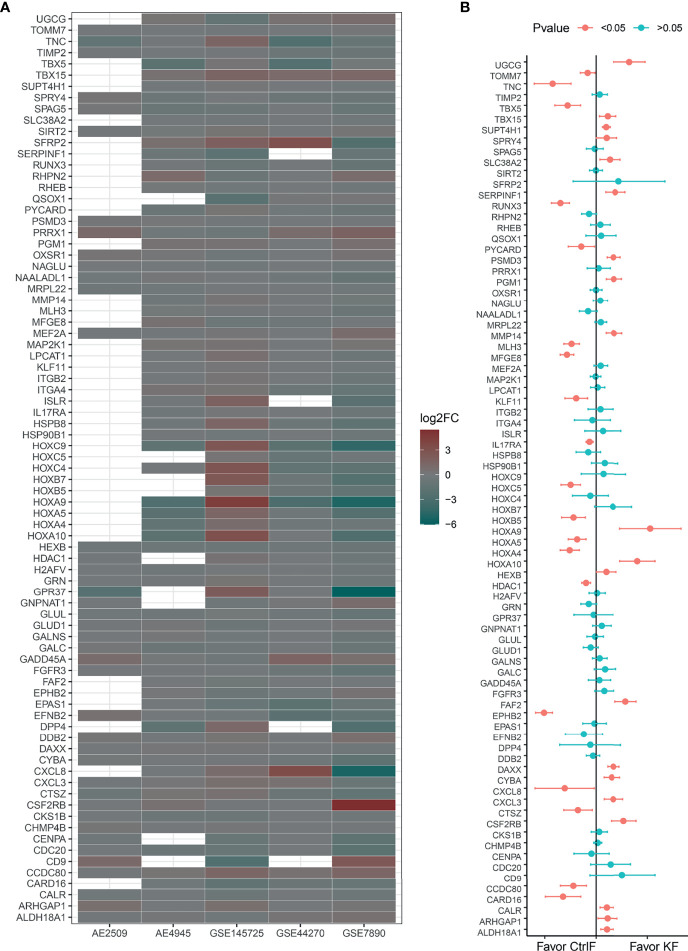
Log2FoldChanges (log2FC) and mean difference of 81 hub DEGs. **(A)** Bubble plot for comparison of the Log2FC between keloid and normal fibroblast of 81 hub DEGs in every data set. Red color indicates a lower expression level in keloid fibroblast as compared with normal skin/scar fibroblast. While green color indicates a higher expression level. Blank dots indicate undetected probes or unmapped entrez gene IDs. **(B)** Estimates fixed effects of mean difference weighted by sample numbers with Hedges’g method for each gene’s group. And 95% confident interval is added at the two side of each own estimated effect size result. Significant P value acquired through two-tail of student’s t-test.

To further understand the roles of the 81 hub DEGs in inflammation and the immune response, GO and KEGG enrichment analyses were performed, and the results are listed in **Tables S1** and **S2**. As shown in **Table S2**, most genes in the biological process category were significantly concentrated in neutrophil-related immunity and reduced in most gene sets (**Figure 3A**).

Fas-associated factor family member 2 (FAF2) plays an important role in regulating the resistance to apoptosis observed in T cells and eosinophils from atopic dermatitis patients (16). Integrin subunit beta 2 (ITGB2) plays an important role in the immune response, including leukocyte adhesion and transmigration of T cells and neutrophils (17), contributes to apoptotic neutrophil phagocytosis by macrophages (18) and is required for CD177-PRTN3-mediated activation of TNF-primed neutrophils (19). Quiescin sulfhydryl oxidase 1 (QSOX1) is induced as fibroblasts begin to exit the proliferative cycle and enter quiescence, and a decreased gene expression level suggests that the fibroproliferative process is activated (20). For the detailed functions of neutrophil-mediated immunity and T cell activation/migration/costimulation-related DEGs, refer to the GeneCards (http://www.genecards.org/) database (21) and **Supplementary Table S3**.

### DEGs Involved in the IL-17 Signaling Pathway Are Abnormally Expressed

The interleukin 17 (IL-17) family, which consists of cytokines IL-17A-F, plays a crucial role in inflammatory responses. Interleukin 17A (IL17A), a proinflammatory cytokine secreted by activated T cells, is a potent inducer of the maturation of CD34^+^ hematopoietic precursors into neutrophils. Interleukin 17A and its receptor IL17RA play a pathogenic role in many inflammatory and autoimmune diseases, such as rheumatoid arthritis (22, 23). CXC motif chemokine ligand 8 (CXCL8/IL8), an inflammatory chemotactic factor induced by activation of IL17RA, attracts neutrophils and T cells in response to an inflammatory stimulus (24). Overproduction of this proinflammatory protein is thought to be associated with cystic fibrosis (25). As shown in the merged result of **Figure 3B**, CXCL8 and IL17RA, which are involved in the IL-17 signaling pathway and fibroblast activation, were expressed at lower levels. At the same time, it can be seen that CXCL3, another member of the CXC chemokine subfamily, usually acts as a neutrophil chemotactic agent in inflammation (26) and is overexpressed in keloid fibroblasts.

### Detection of Local Immune Cell Activity in Keloid Tissue

**Figure 4:**
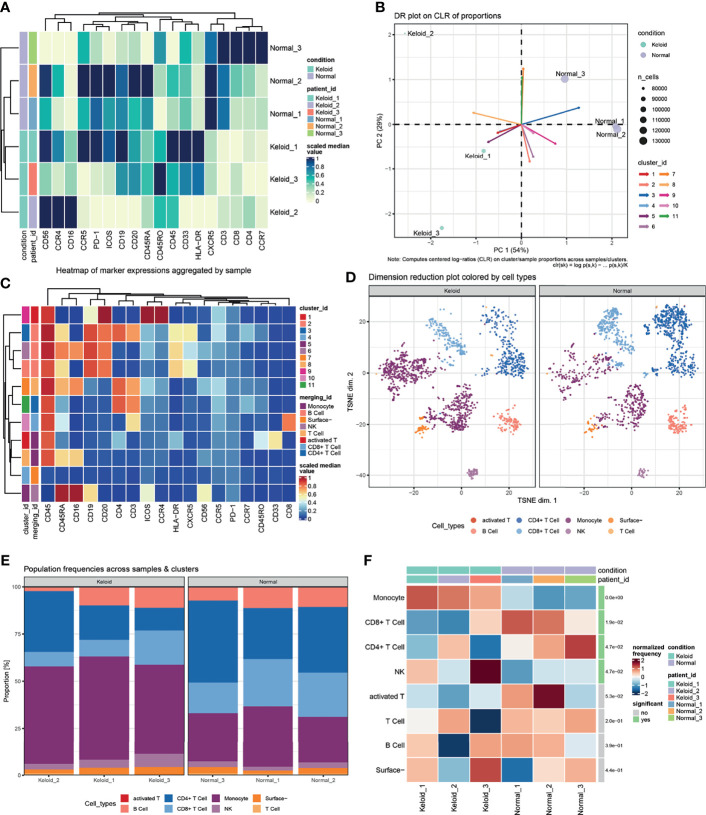
Changes in the proportion of local immune cells between keloid and normal dermal tissue. **(A)** Heatmap of median scaled of marked cells proportion aggregated by samples. **(B)** Computed centered log−ratios (CLR) on cluster/sample proportions for dimension reduction. **(C)** Heatmap of median scaled of marked cells proportion aggregated by clusters or immunocell types. **(D)** Dimension reduction plot colored with artificially annotated cell types. **(E)** Population frequencies of marked cells across samples and immunocell types. **(F)** Significant difference in the normalized frequency of immunocell types between keloid and normal samples.

To further understand the real changes in the local immunological status of keloids, this study performed flow cytometric detection in single-cell suspensions of 3 keloid tissue samples and 3 normal dermal tissue samples. The results are shown in **Figure 4**. As shown in **Figure 4A**, obvious differences in various antibody marked cell proportions are evident between the keloid and normal tissues. After calculating the cumulative distribution function and the number of clusters (**Figure 4B**), 11 clusters were selected as subtypes of marked cells. According to the special types of surface antibodies of different immune cells, 11 clusters were artificially divided into 8 types of cells: monocytes, B cells, NK cells, T cells, activated T cells, CD4+ T cells, CD8+ T cells, and surface cells without antibody labeling (**Figure 4C**). After cluster classification and manual annotation, the differences in the distributions of keloid and normal tissue immune cell clusters can be clearly observed in the dimensionality reduction map based on deep learning (tSNE) (**Figure 4D**). By comparing the proportion of each cell cluster among all collected cells, the proportions of CD4+- and CD8+-labeled T cells from normal tissues were found to be significantly higher than those from keloid tissues, while NK cells and monocytes showed the opposite findings: their proportions were lower in normal tissue samples than in keloid tissue samples (**Figures 4E, F**). Pseudocolor images and histograms of the four cell types (CD3+CD4+, CD3+CD8+, CD33+CD45RO+, and CD16+CD56+) are shown in **Supplementary Figure S4**. In addition, in the process of manual annotation, due to a lack of some antibodies and the uncertainty of subtype cell markers, this study classified uncertain clusters 3 and 6 as B cell populations, and cluster 7 (most likely naive T cells) was classified as a T cell population.

## Discussion

Although the gene chip can identify abnormally expressed genes in diseases, the influence of the accuracy of a single sample on data reliability and the reproducibility of test results cannot be ignored. Therefore, collecting microarray data from multiple platforms and studies can markedly reduce the randomness of the results and facilitate an accurate understanding of abnormal gene expression and biological process changes in diseases. We focused on studying 81 core DEGs with highly identical results and abnormal expression across multiple datasets and further analyzing potential candidates for targeted therapy.

Chronic inflammation and the immune response have been established to have critical roles in wound healing and keloid formation (27, 28). The results of the preliminary screening analysis identified three genes: NEO1, SKAP2 and BPGM. NEO1 (neogenin 1) encodes a cell surface protein belonging to the immunoglobulin superfamily and has been identified to be involved in cell growth and differentiation and in cell-cell adhesion (29). SKAP2 (Src kinase-associated phosphoprotein 2) encodes an adaptor protein that is thought to play an essential role in the Src signaling pathway and is required for defense against *K. pneumoniae* infection and neutrophil respiratory burst (30). Then, the hallmarks of the biological states or processes of the 81 hub DEGs were found to enhance their critical roles in inflammation and the immune response.

Although keloids are not malignant tumors, the pain caused by their continuous overgrowth and the high recurrence rate warrant consideration. Recently, a few T cell subtypes (e.g., T-reg, Th1, Th2 and Th17/Th22 cells) have been identified to play critical roles in the pathological mechanisms of keloid formation (31, 32). Consequently, immune infiltration enrichment with the GSVA method for DEGs was conducted in this study, and the results mainly focused on T cell subtypes (e.g., Th1, Th2, Th17, and T-reg cells), followed by NK cells (e.g., CD56dim NK, CD56bright NK and NK cells). The CyTOF data showed that the ratios of CD4+ T cells, CD8+ T cells and NK cells in keloid tissue were significantly different from those in normal dermal skin. Furthermore, Pearson correlations between groups also demonstrated the crucial roles of T cell subtypes (e.g., Th17, CD8Tcm and T follicular cells). Thus, T cells are critical to keloid formation.

A recent study showed that significant Th17 cell infiltration occurs in the marginal area of keloid tissue fibroblasts, and a local increase in IL17 can induce stromal cell-derived factor-1 (SDF-1) to indirectly recruit Th17 cells and create a positive feedback loop (33). In addition, SDF-1 also stimulates the production of collagen and α-SMA. In addition to the evidence indicating that IL-17-producing Th17 cells stimulate keloid formation, we also found Th2 cells producing IL-4 and IL-13 in the immune infiltrates, which are considered important mediators in the pathogenesis of fibroproliferative diseases such as scleroderma-like cutaneous syndromes (34), abnormal scarring (35) and keloid formation (36). Furthermore, a recent cross-sectional study by Nangole et al. (37) showed that IL-4, IL-13 and a variety of other cytokines were significantly elevated in the plasma of keloid patients compared with normal subjects, leading the authors to speculate that the immune system plays a role similar to that of autoinflammatory disease in the formation of keloid. Among the DEGs involved in the IL-13 signaling pathway identified by the KEGG analysis, CXCL8 was recently confirmed to play an important role in keloid scar formation (38, 39). However, the pathological mechanism of ITGB2 and HSP90B1 in keloid formation is still unknown.

**Figure 5:**
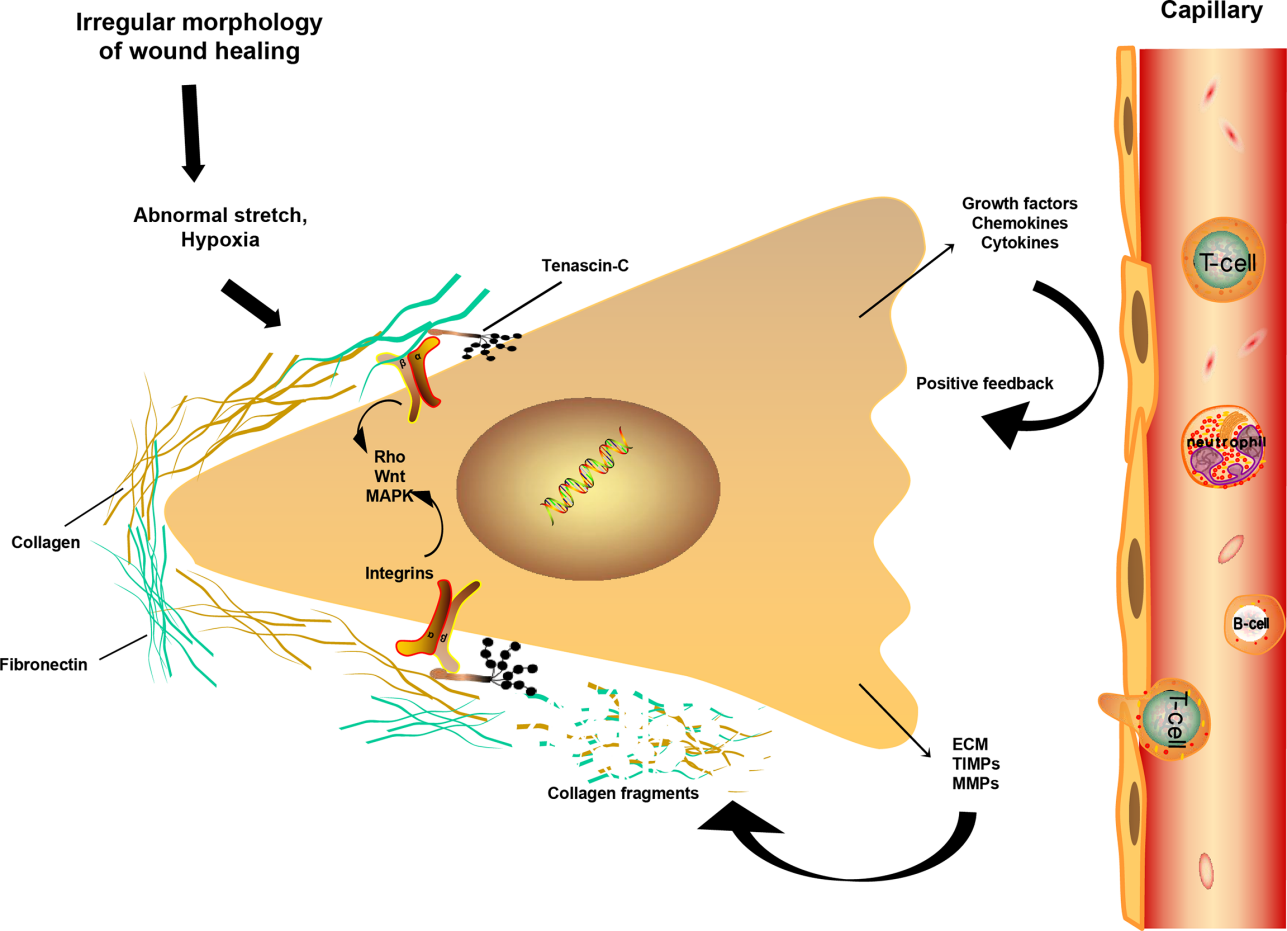
Fibrosis mechanism of fibroblast during keloid formation. Schematic drawing of a fibroblast and its mechanical interaction with ECM. Under continuous stress and strain and low oxygen environment, the disappearance of the temporal protein TNC increases the interaction of integrins with ECM collagen or fibronectin. Fibroblasts are further activated, releasing more chemokines and growth factors, leading to an increase in ECM and fibrosis. At the same time, the increase of TIMPs and MMPs degrades collagen and fibronectin, forming a positive circulation, resulting in the formation and continuous increase of keloid, even invading the surrounding normal tissues. Main signaling pathways associated with integrins activation include the Rho, WNT and MAPK pathways.

Apart from the findings discussed above, tenascin-C (TNC), a DEG identified in our analysis that participates in ECM organization pathways, encodes ECM proteins and contributes to tissue homeostasis and wound healing (40), but also to acute inflammation (41) and keloid fibroblast activity (42). Notably, TNC shows obvious temporal and spatial expression, and several reports have shown that IL-4 (43) and IL-13 (44) can directly induce its production. Another DEG involved in ECM breakdown is matrix metallopeptidase 14 (MMP14), which acts as a positive regulator of cell growth in wound healing during keratinocyte migration induced by TGF-β (45). Deposition of collagen and ECM, the main constituents of keloids, results from overexpression of growth factors and cytokines (46). The other two DEGs involved in ECM degradation are the integrins alpha-4 and beta-2 (ITGA4 and ITGB2), which are important cell surface receptors that mediate the binding of cell adhesion molecules and fibronectin to ECM, forming a mechanically sensitive structure with a key role in mechanical transduction (47, 48). The schematic diagram in **Figure 5** shows a continuous cycle of fibroblast fibrosis and proliferation during keloid formation, which helps elucidate the interaction between the extracellular and internal environments of keloid formation.

Although the best strategies to treat keloids have been found through genomic analysis, drug trials and animal models, to date, no effective anti-scar therapy can restore the original shape of the injured part, which warrants consideration. Even the use of healthy skin transplantation will result in scars, and a possibility of failure exists. At present, the commonly used method for keloid treatment is scar revision and postoperative inhibition of inflammation, angiogenesis and collagen production (46) using corticosteroids, cryotherapy, radiotherapy, laser therapy, 5-fluorouracil, interferon,bleomycin, etc. In addition to these therapeutic methods that have been applied, more effective therapies or drugs to relieve keloid patients’ suffering are urgently needed. For example, brodalumab, an IL17RA antagonist, has been used to treat moderate to severe plaque psoriasis (49); miglustat, a glycosphingolipid synthesis inhibitor, has been used in diseases in which the enzyme glucocerebrosidase is deficient, such as Gaucher disease (50) and cystic fibrosis (51); and elosulfase alfa helps correct N-acetylglucosamine-6-sulfate sulfatase enzyme deficiency in mucopolysaccharidosis type IV (52). However, none of these drugs have been applied to treat keloids. These results provide important insight for further exploration of keloid treatments in the future. Although these drugs showed potential significance for keloid treatment in our previous data analysis, a large number of cell samples and clinical trials are still needed for further verification and confirmation.

In conclusion, this study reinforces the involvement of several immune cell subtypes and genes in fibrosis-related immune response pathways, including the IL-17, IL-4 and IL-13 signaling pathways, as well as ECM organization and degradation processes, providing key evidence for epigenetic keloid genes, deepening our understanding of changes in the keloid fibroblast process, and revealing the key candidate genes affecting keloid formation. This study enhances the preliminary understanding of the influence of immune cell infiltration on keloid formation and will help elucidate the molecular basis of abnormal wound healing to facilitate exploration of better and more effective strategies to limit the occurrence of wound hyperfibrosis and keloid recurrence.

## Data Availability Statement

Publicly available data sets were analyzed in this study. This data can be found here: Data sets related to this article were acquired form GEO (GSE7980, GSE44270, and GSE145725) and ArarryExpress (E-MTAB-2509 and E-MTAB-4945). The original data of the flow cytometry tests are deposited in the public repository FlowRepository, reference ID: FR-FCM-Z4P4.

## Ethics Statement

The studies involving human participants were reviewed and approved by the Medical Ethics Committee of Qingdao Municipal Hospital. The patients/participants provided their written informed consent to participate in this study. Written informed consent was obtained from the individual(s) for the publication of any potentially identifiable images or data included in this article.

## Author Contributions

Conceptualization: YL, ML. Data collection and analysis: CQ, YXL, ZT, XW. Methodology: ZZ, ZY. Visualization: YL, LX. Writing: YL, ML, CQ. Review and editing: TS, YXL, ZT. All authors contributed to the article and approved the submitted version.

## Funding

This work is partially supported by the Class B of Qingdao Medical and Health Key Discipline Construction Project:2020-2023 and Qingdao Medical and Health Outstanding Young Talent Training Project:2020-2023. The funders had no role in study design, data collection and analysis, decision to publish, or preparation of the manuscript.

## Conflict of Interest

The authors declare that the research was conducted in the absence of any commercial or financial relationships that could be construed as a potential conflict of interest.

## Publisher’s Note

All claims expressed in this article are solely those of the authors and do not necessarily represent those of their affiliated organizations, or those of the publisher, the editors and the reviewers. Any product that may be evaluated in this article, or claim that may be made by its manufacturer, is not guaranteed or endorsed by the publisher.

## Supplementary Material

Supplementary Figure S1 Venn diagram of DEGs in each study group.

Supplementary Figure S2 Pearson correlation of tumor estimated immune scores and immune cell GSVA z-scores of each samples between keloid and normal skin/scar fibroblast.

Supplementary Figure S3 Hallmarks heatmap of 81 DEGs’ biological states or process.

Supplementary Figure S4 Pseudocolor and histograms of cell types (CD3+CD4+, CD3+CD8+, CD33+CD45RO+, and CD16+CD56+).

Supplementary Table S1 Gene ontology results of 81 hub DEGs.

Supplementary Table S2 KEGG pathway analysis results of 81 hub DEGs.

Supplementary Table S3 Detailed function of important genes.

Supplementary Table S4 Fibroblast growth factor receptor associated publications.

Supplementary Table S5 Antibody list for Mass Cytometry.
