# Supplementary material for: The Polygenic Map of Keloid Fibroblasts Reveals Fibrosis-Associated Gene Alterations in Inflammation and Immune Responses
Source: Front Immunol. 2022 Jan 10;12:810290. doi: 10.3389/fimmu.2021.810290 (PMC8785650; doi:10.3389/fimmu.2021.810290)
Supplement: Supplementary Figure S1 — Venn diagram of DEGs in each study group. [file Image_1.pdf]

Figure S1

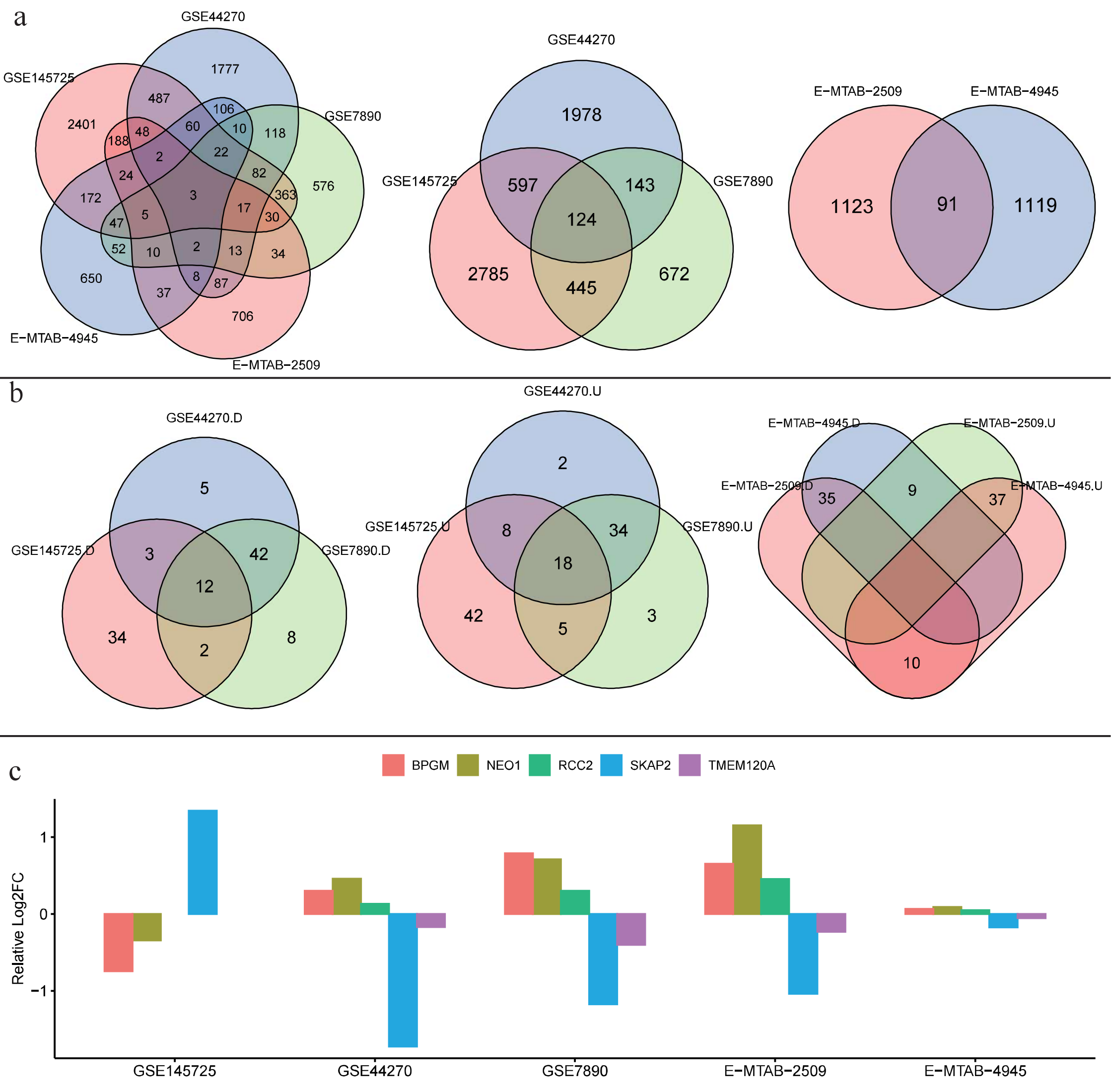

Figure S1. Venn diagram of DEGs in each study group.

a: Venn diagrams of DEGs in all datasets, and Venn diagrams of DEGs in GEO and AE datasets.

b: Venn diagrams of up-regulated and down-regulated DEGs of GEO and AE datasets.

c: The relative expression differences of the shared genes BPGM, NEO1, RCC2, SKAP2 and TMEM120A in each data set.
