# Supplementary material for: The Polygenic Map of Keloid Fibroblasts Reveals Fibrosis-Associated Gene Alterations in Inflammation and Immune Responses
Source: Front Immunol. 2022 Jan 10;12:810290. doi: 10.3389/fimmu.2021.810290 (PMC8785650; doi:10.3389/fimmu.2021.810290)
Supplement: Supplementary Figure S2 — Pearson correlation of tumor estimated immune scores and immune cell GSVA z-scores of each samples between keloid and normal skin/scar fibroblast. [file Image_2.pdf]

Figure S2

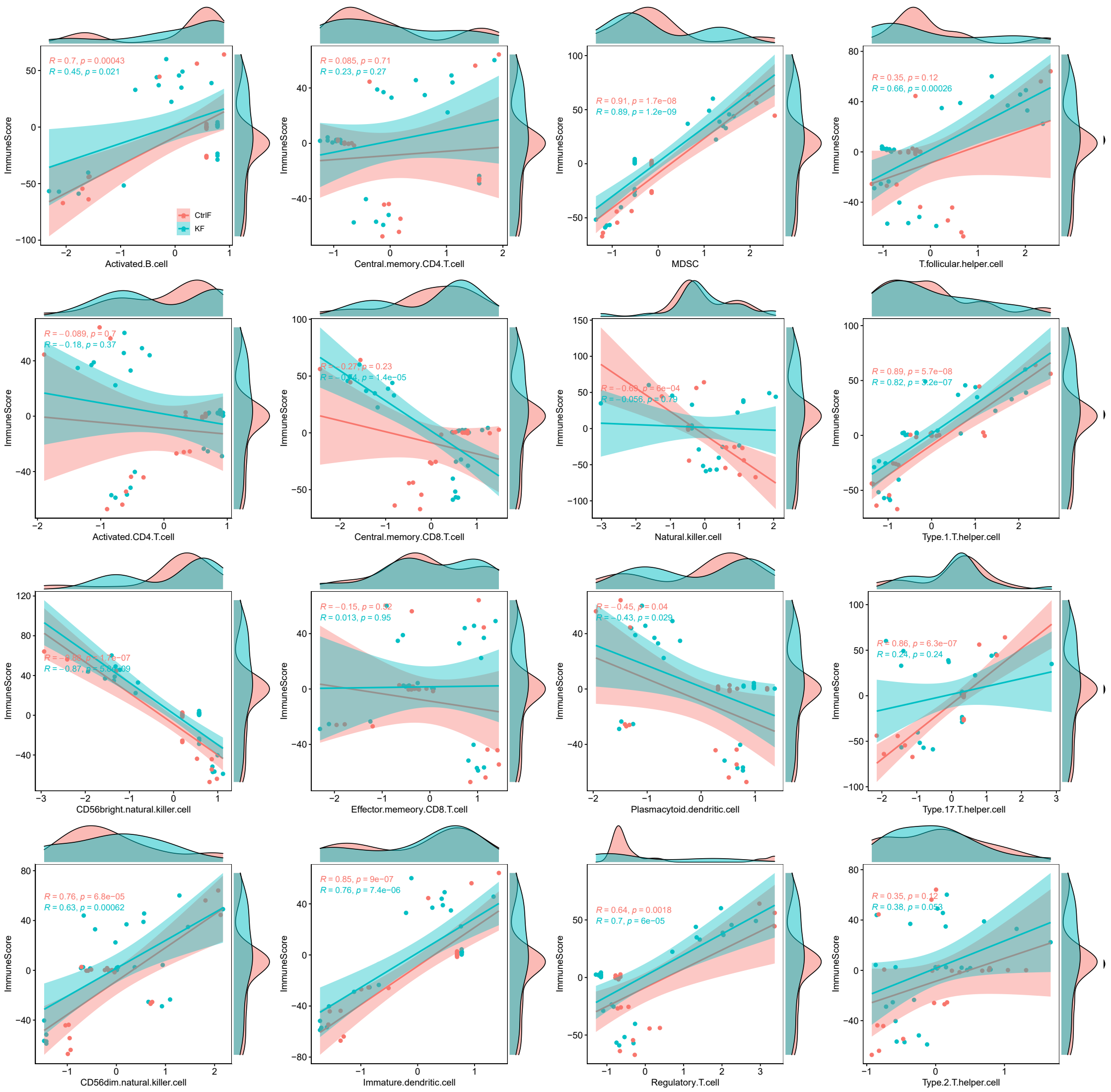

Figure S2: Pearson correlation of tumor estimated immune scores and immune cell GSVA z-scores of each samples between keloid and normal skin/scar fibroblast. (KF: keloid fibroblast, CtrlF: normal skin/scar fibroblast)
