# Supplementary material for: The Polygenic Map of Keloid Fibroblasts Reveals Fibrosis-Associated Gene Alterations in Inflammation and Immune Responses
Source: Front Immunol. 2022 Jan 10;12:810290. doi: 10.3389/fimmu.2021.810290 (PMC8785650; doi:10.3389/fimmu.2021.810290)
Supplement: Supplementary Figure S3 — Hallmarks heatmap of 81 DEGs’ biological states or process. [file Image_3.pdf]

Figure S3

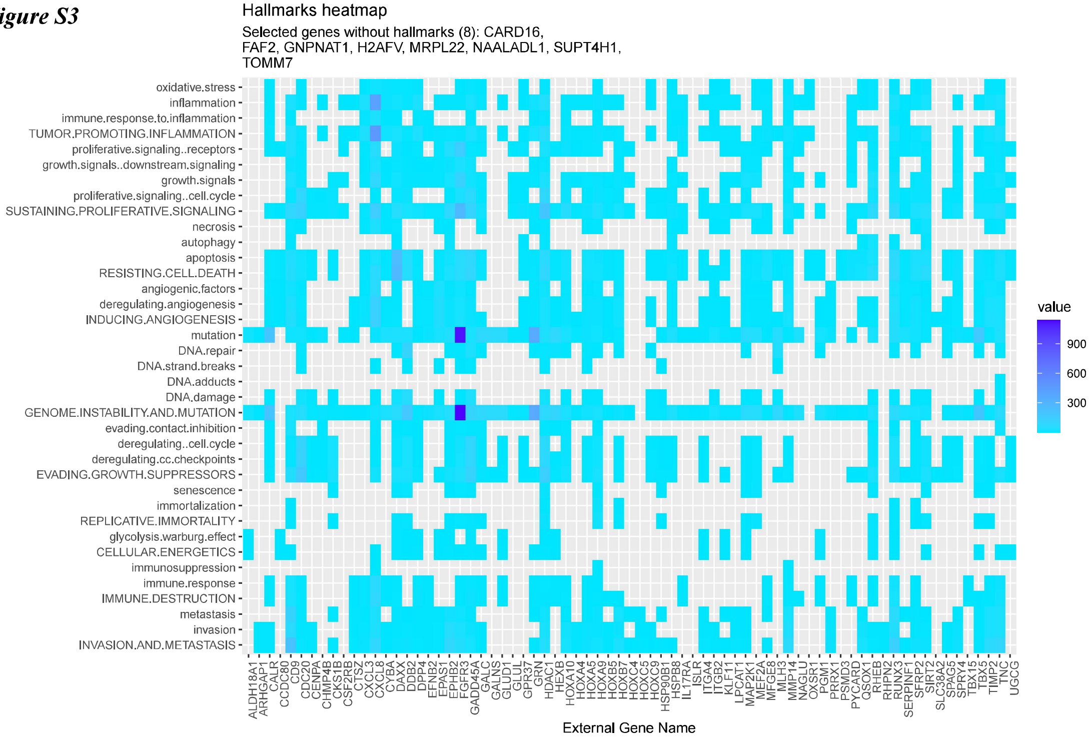

Figure S3. Hallmarks heatmap of 81 DEGs' biological states or processes.

(Hallmarks of cancer count of 81 hub DEGs retrieved through a text mining approach, which is designed to organize and evaluate scientific literature on cancer.)
