# Supplementary material for: The Polygenic Map of Keloid Fibroblasts Reveals Fibrosis-Associated Gene Alterations in Inflammation and Immune Responses
Source: Front Immunol. 2022 Jan 10;12:810290. doi: 10.3389/fimmu.2021.810290 (PMC8785650; doi:10.3389/fimmu.2021.810290)
Supplement: Supplementary Figure S4 — Pseudocolor and histograms of cell types (CD3+CD4+, CD3+CD8+, CD33+CD45RO+, and CD16+CD56+). [file Image_4.pdf]

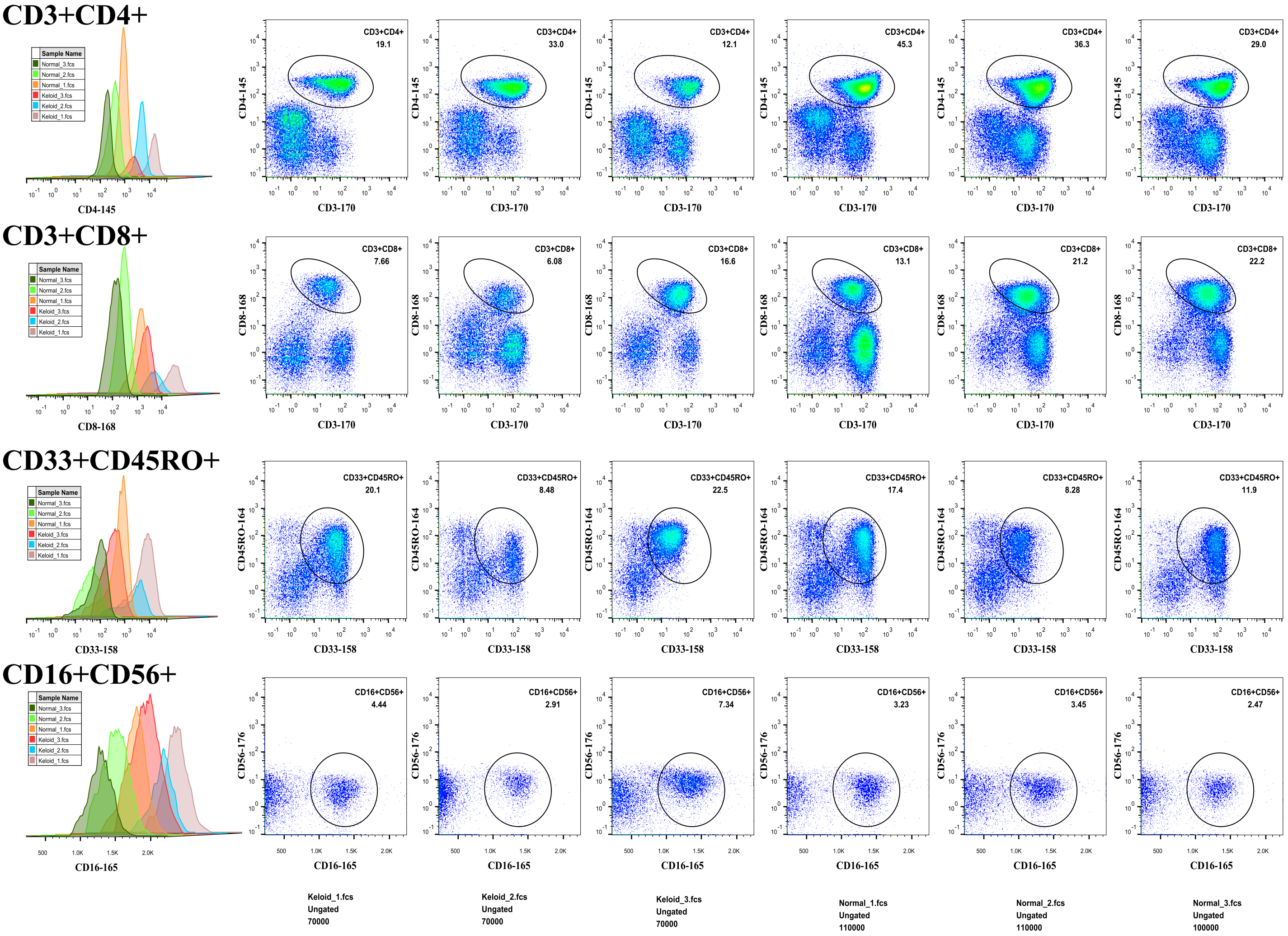

Figure S4. Pseudocolor and histograms of the significant difference in normalized frequency of immunocell types between keloid and normal samples.
